# 625 Patient Centered Depression and Anxiety Screening in an Adult Burn Clinic

**DOI:** 10.1093/jbcr/irac012.253

**Published:** 2022-03-23

**Authors:** Jodi Wojcik

**Affiliations:** UofL Health- University Hospital, anchorage, Kentucky

## Abstract

**Introduction:**

Depression and anxiety are seen in burn patients at rates up to 45%. Untreated, they can lead to long-term complications of posttraumatic stress disorder. Treating the psychological consequences of burn injury leads to increased quality of life for patients.

**Methods:**

Rapid-cycle quality improvement using four plan-do-study-act cycles was used to evaluate interventions. Reviewing the data helped guide new tests of change each cycle. Data were analyzed using run charts to assess each intervention's impact.

**Results:**

A medical screening rate of 100% was achieved. Thirty three percent of patients screened were positive for depression and/or anxiety. Of the patients who screened positive, 100% chose an intervention on the Shared Decision Making Tool.

**Conclusions:**

Universal screening increases recognition of anxiety and depression in a vulnerable population of burn patients. Using a Shared Decision Making tool increases patients centerdness and selecting an intervention for treatment.

